# The targets of aspirin in bladder cancer: bioinformatics analysis

**DOI:** 10.1186/s12894-022-01119-z

**Published:** 2022-10-31

**Authors:** Xiao Li, Yanghao Tai, Shuying Liu, Yating Gao, Kaining Zhang, Jierong Yin, Huijuan Zhang, Xia Wang, Xiaofei Li, Dongfeng Zhang, Dong-feng Zhang

**Affiliations:** 1 Department of Thoracic Oncology, Lin Fen Central Hospital, 041000 Lin Fen, China; 2 Shanxi Medical University, 030000 Taiyuan, China

**Keywords:** Aspirin, Bladder cancer, Target gene, Comprehensive bioinformatics analysis

## Abstract

**Background:**

The anti-carcinogenic properties of aspirin have been observed in some solid tumors. However, the molecular mechanism of therapeutic effects of aspirin on bladder cancer is still indistinct. We introduced a bioinformatics analysis approach, to explore the targets of aspirin in bladder cancer (BC).

**Methods:**

To find out the potential targets of aspirin in BC, we analyzed direct protein targets (DPTs) of aspirin in Drug Bank 5.0. The protein-protein interaction (PPI) network and signaling pathway of aspirin DPTs were then analyzed subsequently. A detailed analysis of the KEGG (Kyoto Encyclopedia of Genes and Genomes) pathway has shown that aspirin is linked to BC. We identified overexpressed genes in BC comparing with normal samples by Oncomine and genes that interlinked with aspirin target genes in BC by STRING.

**Results:**

Firstly, we explored 16 direct protein targets (DPT) of aspirin. We analyzed the protein-protein interaction (PPI) network and signaling pathways of aspirin DPT. We found that aspirin is closely associated with a variety of cancers, including BC. Then, we classified mutations in 3 aspirin DPTs (CCND1, MYC and TP53) in BC using the cBio Portal database. In addition, we extracted the top 50 overexpressed genes in bladder cancer by Oncomine and predicted the genes associated with the 3 aspirin DPTs (CCND1, MYC and TP53) in BC by STRING. Finally, 5 exact genes were identified as potential therapeutic targets of aspirin in bladder cancer.

**Conclusion:**

The analysis of relevant databases will improve our mechanistic understanding of the role of aspirin in bladder cancer. This will guide the direction of our next drug-disease interaction studies.

## Background

It is universally acknowledged that bladder cancer (BC) is one of the most common cancers, and most bladder cancers are uroepithelial, with approximately 75% of patients affected by non-muscle invasive bladder cancer (NMIBC) [1]. According to the World Health Organization (WHO), the number of BC cases and deaths is expected to increase in the future [2, 3]. Bladder cancer occurs in two different pathways originating from superficial bladder cancer (SBC), NMIBC, and muscle-invasive bladder cancer (MIBC). For many years, the treatment of BC was limited to surgery and immunotherapy or chemotherapy. In recent years, studies on genetic analysis have guided new therapeutic approaches [4]. The FDA approved the use of the programmed death receptor ligand 1 (PD-L1) inhibitors atezolizumab and avelumab, as well as the PD-1 inhibitors nivolumab and pembrolizumab, in patients with advanced or metastatic uroepithelial cancer [5–7].

Aspirin is a powerful antiplatelet agent widely used in patients with coronary atherosclerosis [8]. guidelines published by the USPSTF affirm the benefit of aspirin in the prevention of colorectal cancer [9]. In recent years, the anticancer properties of aspirin have been observed in some solid tumors, such as prostate cancer [10], pancreatic cancer [11], and endometrial cancer [12]. However, the mechanism of action related to aspirin in bladder cancer is still unclear. In recent years, many genomic studies have identified numerous cancer-related genes, providing insight into the molecular mechanisms of disease progression. Databases combining drug data with target and drug action information have also continued to improve. The integrated analysis of cancer-associated action genes and drugs provides a good opportunity to discover new targets for drug development. A better understanding of the molecular mechanisms of pharmacological action may lead to the discovery of new applications of existing drugs.

In this study, we used bioinformatics to perform relevant analyses. First, we searched for direct protein targets (DPT) of aspirin in DrugBank. The protein interaction (PPI) network and signaling pathways of aspirin DPT were then analyzed. Analysis of the KEGG (Kyoto Encyclopedia of Genes and Genomes) pathway showed that aspirin is associated with a variety of cancers, including bladder cancer. Next, we screened 3 genes (CCND1, MYC and TP53) as the most important target genes of aspirin in BC. Mutations in these 3 genes were analyzed using cBioPortal. Meanwhile, we compared tumors with normal groups by Oncomine to identify overexpressed genes in BC and used STRING method to find genes associated with 3 target genes of aspirin in BC. Finally, we identified 5 target genes as potential therapeutic targets for aspirin in BC. Further KEGG pathway analysis of these 5 target genes confirmed our predictions. We believe that these findings contribute to the understanding of the mechanism of aspirin in BC and provide us with a new approach to analyze the targets and mechanisms of aspirin and even other drugs.

## Methods

### Identification of aspirin direct protein targets (DPT)

Drugbank (https://go.drugbank.com/) is an annotated rich resource that combines detailed drug data with comprehensive drug target and action information. Since its initial release in 2006, DrugBank has been used extensively to facilitate electronic drug target discovery, drug design, drug docking or screening, drug metabolism prediction, drug interaction prediction, and general pharmacy education. A wealth of drug metabolomics, drug proteomics, and drug transcriptomics information has been compiled from the primary literature [13]. The direct protein target (DPT) of aspirin was obtained from Drugbank.

### 2.2 Protein-protein interaction (PPI) networks and signaling pathways analyzed by aspirin DPTs

STRING (https://string-db.org/) is an online tool designed to evaluate protein-protein interaction (PPI) networks. Cytoscape is an open-source software project for integrating biomolecular interaction networks with high-throughput expression data and other molecular states into a unified conceptual framework [14]. CluePedia, a plug-in for Cytoscape software, is a tool to search for potential genes associated with specific signaling pathways by calculating linear and nonlinear statistical correlations of experimental data [15]. The PPI network of aspirin DPTs consists of STRING with a cutoff criterion of no more than 20 interactors in layers 1 and 2. We also analyzed the signaling pathways of aspirin DPTs by STRING, and then validated and visualized them by CluePedia. And the KEGG pathway of aspirin target genes in bladder cancer was identified by CluePedia. p < 0.05 was set as the cut-off criterion for pathway enrichment analysis.

### Exploring genomic data of aspirin DPT in BC

The cBioPortal (https://cbioportal.org) is an open platform in which we can explore multidimensional cancer genomics data. OncoPrint is a tool that allows visualization of changes in tumor samples in gene arrays [16]. With cBioPortal and OncoPrint, we explored and visualized alterations in 3 aspirin DPTs (CCND1, MYC, and TP53) associated with BC, and the frequency of genomic alterations in the selected cancer studies was used as a filter.

### Identification of potential therapeutic target genes for aspirin in BC

Oncomine (https://www.oncomine.org) is a cancer microarray database and web-based data mining platform for facilitating discovery of genome-wide expression analysis. Differential gene expression is identified by comparing major types of cancer with corresponding normal tissues [17]. Oncomine was used to define overexpressed genes in BC, select BC as the cancer type, and select cancer versus normal analysis as the type of analysis for screening. Also, genes associated with 3 aspirin DPT in BC were predicted by STRING and visualized by Cytoscape. The above two data sets were crossed to obtain potential therapeutic target genes for aspirin in BC. A cut-off value of p < 0.05 was used.

### Gene Ontology analysis of potential therapeutic target genes for aspirin in bladder cancer

Gene Ontology (GO; http://www.geneontology.org/) is a community-based bioinformatics resource that uses ontologies to represent biological knowledge and provide information about the function of gene products [18]. GO analysis was used to determine the functional annotation of potential therapeutic target genes for aspirin in BC. First, Homo sapiens was used as a biological filter. Next, biological processes, molecular functions, or cellular components were set as GO-level filters. Then, select the data provided by GO_Central. Finally, we confirmed the first GO term as the most important function of potential therapeutic target genes of aspirin in BC.

## Results

### Identification of aspirin DPT

In the DrugBank output, aspirin is described as an analgesic, non-narcotic anti-inflammatory agent, non-steroidal anti-inflammatory agent, antipyretic agent, anti-rheumatic agent, cardiovascular agent, central nervous system agent, cyclooxygenase inhibitor, platelet aggregation inhibitor, and sensory system agent. We identified a total of 16 major direct protein targets (DPT) of aspirin including PTGS1, PTGS2, AKR1C1, PRKAA1, EDNRA, TP53, HSPA5, RPS6KA3, NFKBIA, TNFAIP6, CASP1, CASP3, CCND1, MYC, PCNA, NEU1 (Table 1).

**Table 1:** Identification of direct targets of aspirin using DRUGBANK.

DB_ID	Name	Gene name	Uniprot ID	Action
DB00945	Acetylsalicylic acid	PTGS1	P23219	Inhibitor
		PTGS2	P35354	Inhibitor
		AKR1C1	Q04828	Inhibitor
		PRKAA1	Q13131	Activator
		EDNRA	P25101	Inhibitor
		TP53	P04637	Inducer
		HSPA5	P11021	Inhibitor Binder
		RPS6KA3	P51812	Inhibitor
		NFKBIA	P25963	Inhibitor
		TNFAIP6	P98066	Inhibitor Downregulator
		CASP1	P29466	Inhibitor Downregulator
		CASP3	P42574	Inhibitor Downregulator
		CCND1	P24385	Inhibitor
		MYC	P01106	Inhibitor
		PCNA	P12004	Inhibitor
		NEU1	Q99519	Inhibitor

### Direct association of aspirin with bladder cancer

**Fig. 1:**
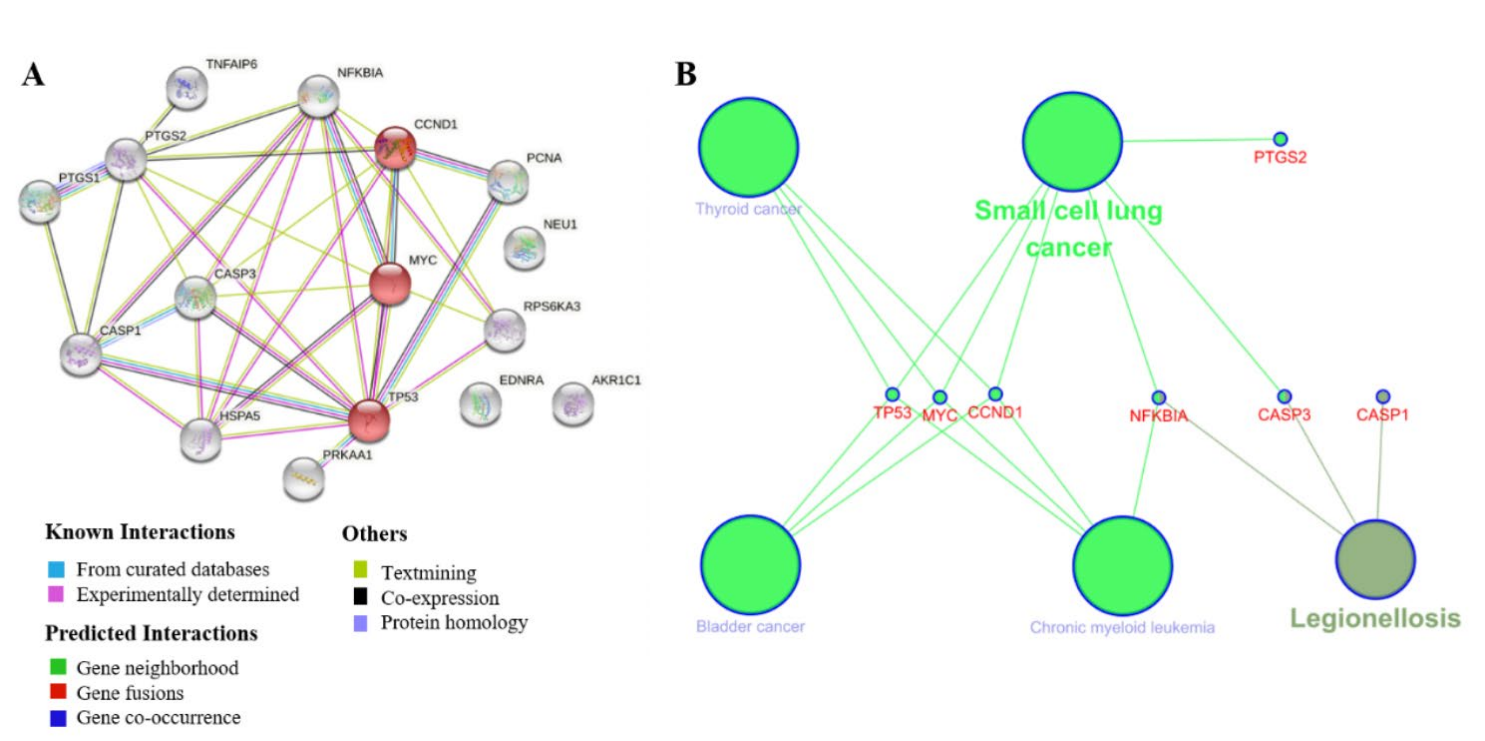
Protein-protein interaction (PPI) network of 16 DPTs of aspirin. **A** KEGG signaling pathway of aspirin 16 DPTs. The 3 nodes in red color are related to bladder cancer. **B** The visualization of PPI network using CluePedia.

The PPI network and signaling pathways of 16 aspirin DPTs were generated by STRING (Table 2). the top 5 KEGG pathways of DPTs were thyroid cancer, bladder cancer, small cell lung cancer, legionellosis, and chronic granulocytic leukemia. The results showed that aspirin was associated with bladder cancer (p = 0.00014) and three aspirin DPTs (CCND1, MYC, and TP53) were associated with BC (Table 2). We then validated and visualized the association of aspirin with BC by CluePedia (Fig. 1B). The three DPTs associated with BC by aspirin were consistent with the results of STRING (Fig. 1).

**Table 2:** Top 5 KEGG pathways associated with the DPTs of aspirin

pathway	description	count	false discovery rate	Gene ID
hsa05216	Thyroid cancer	3	0.00011	TP53、CCND1、MYC
hsa05219	Bladder cancer	3	0.00014	TP53、CCND1、MYC
hsa05222	Small cell lung cancer	6	3.48E-08	PTGS2、NFKBIA、CASP3、TP53、CCND1、MYC
hsa05134	Legionellosis	3	0.00029	NFKBIA、CASP1、CASP3
hsa05220	Chronic myeloid leukemia	4	1.68E-05	TP53、CCND1、MYC、NFKBIA

### Genetic alterations of aspirin DPTs in patients with bladder cancer

**Fig. 2:**
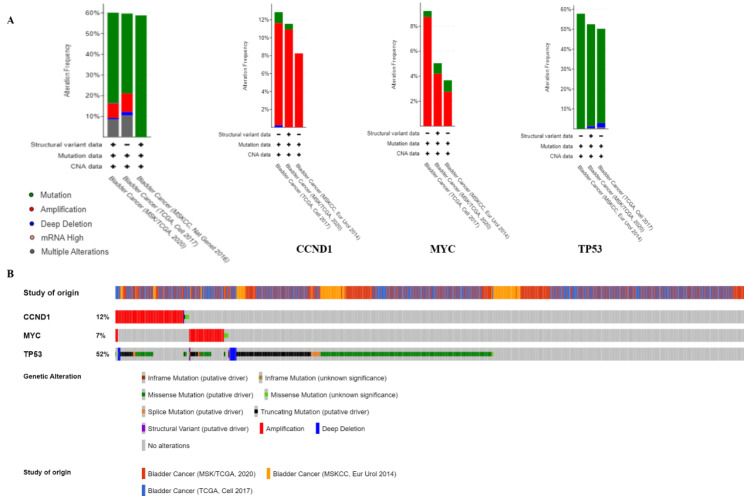
Analysis of genetic alterations of 3 DPTs of aspirin (CCND1, MYC and TP53) in human bladder cancer studies embedded in cBio cancer genomics portal. **A** Overview of the changes in CCND1, MYC and TP53 in genomics datasets available in 3 different studies. **B** Visual summary of mutations across a set of human bladder cancer samples based on a query of the 3 genes in OncoPrint. Distinct genomic alterations including mutations, copy number alterations, exemplified by gene amplifications and homozygous deletions, are summarized and color coded (as % changes) in the affected genes in individual tumor samples. Each row represents a gene, and each column represents a tumor sample

To understand the expression and function of the three aspirin DPTs associated with BC (CCND1, MYC and TP53), the genetic alterations of these genes in BC patients were explored through the cBioportal. We examined cancer genomic alterations and clinical expression characteristics of CCND1, MYC and TP53 in bladder cancer. We obtained a summary of the polygenic alterations by studying MSKCC, TCGA, and MSK/TCGA. The most significant genomic alterations were presented using OncoPrint. The results showed that 646 cases (70%) had at least one alteration in CCND1, MYC and TP53 (Fig. 2). alterations in CCND1 were presented as gene amplification. alterations in MYC were mainly presented as gene amplification. alterations in TP53 mainly included missense mutations, splice mutations and truncation mutations (Fig. 2).

### Prediction of BC-associated genes with 3 aspirin DTPs

**Fig. 3:**
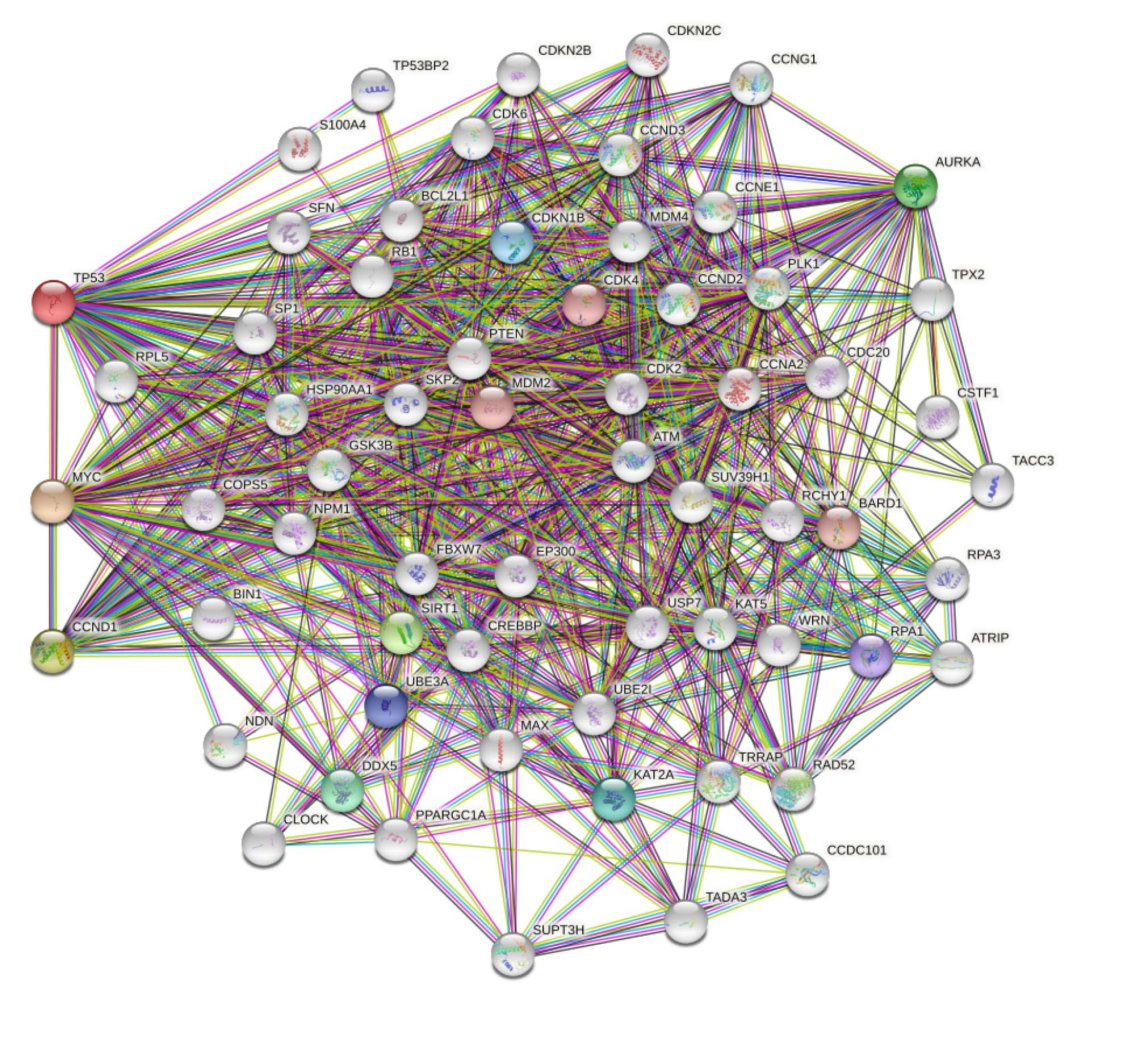
Visual display of the gene network connected to CCND1, MYC and TP53.

Based on the information in the STRING database, the PPI network of genes and the 3 aspirin DPTs in BC were obtained (Fig. 3), and we identified many aspirin-related genes. KEGG pathway analysis showed that these genes are mainly involved in Cell cycle, Human T-cell leukemia virus 1 infection, Viral carcinogenesis, p53 signaling pathway, and Pathways in cancer, etc. (Table 3).

**Table 3:** TOP 5 KEGG pathways in the PPI of the DPTs of aspirin

term description		count	strength	false discovery rate	GENE ID
Cell cycle		23	1.77	3.61E-31	CCND1, CDKN1B, CDK4, MDM2, CCND2, CREBBP, CCNE1, CDKN2C, EP300, CDK6, CDK2, RB1, TP53, CCNA2, SKP2, CDKN2B, ATM, PLK1, GSK3B, SFN, CDC20, CCND3, MYC
Human T-cell leukemia virus 1 infection		21	1.49	5.40E-23	KAT2A, CCND1, CDK4, CCND2, CREBBP, CCNE1, CDKN2C, EP300, CDK2, RB1, TP53, CCNA2, CDKN2B, ATM, BCL2L1, KAT5, TRRAP, PTEN, CDC20, CCND3, MYC
Viral carcinogenesis		20	1.53	9.44E-23	KAT2A, CCND1, CDKN1B, UBE3A, CDK4, MDM2, CCND2, CREBBP, CCNE1, EP300, CDK6, CDK2, RB1, TP53, CCNA2, SKP2, CDKN2B, USP7, CDC20, CCND3
p53 signaling pathway		16	1.84	2.22E-22	CCND1, CDK4, MDM2, CCND2, CCNE1, CDK6, CDK2, TP53, ATM, BCL2L1, RCHY1, SFN, CCNG1, MDM4, PTEN, CCND3
Pathways in cancer		23	1.14	2.48E-18	CCND1, CDKN1B, CDK4, MDM2, CCND2, CREBBP, CCNE1, EP300, CDK6, CDK2, RB1, TP53, CCNA2, SKP2, CDKN2B, BCL2L1, GSK3B, SP1, HSP90AA1, MAX, PTEN, CCND3, MYC

### Identification of overexpressed genes in bladder cancer

**Fig. 4:**
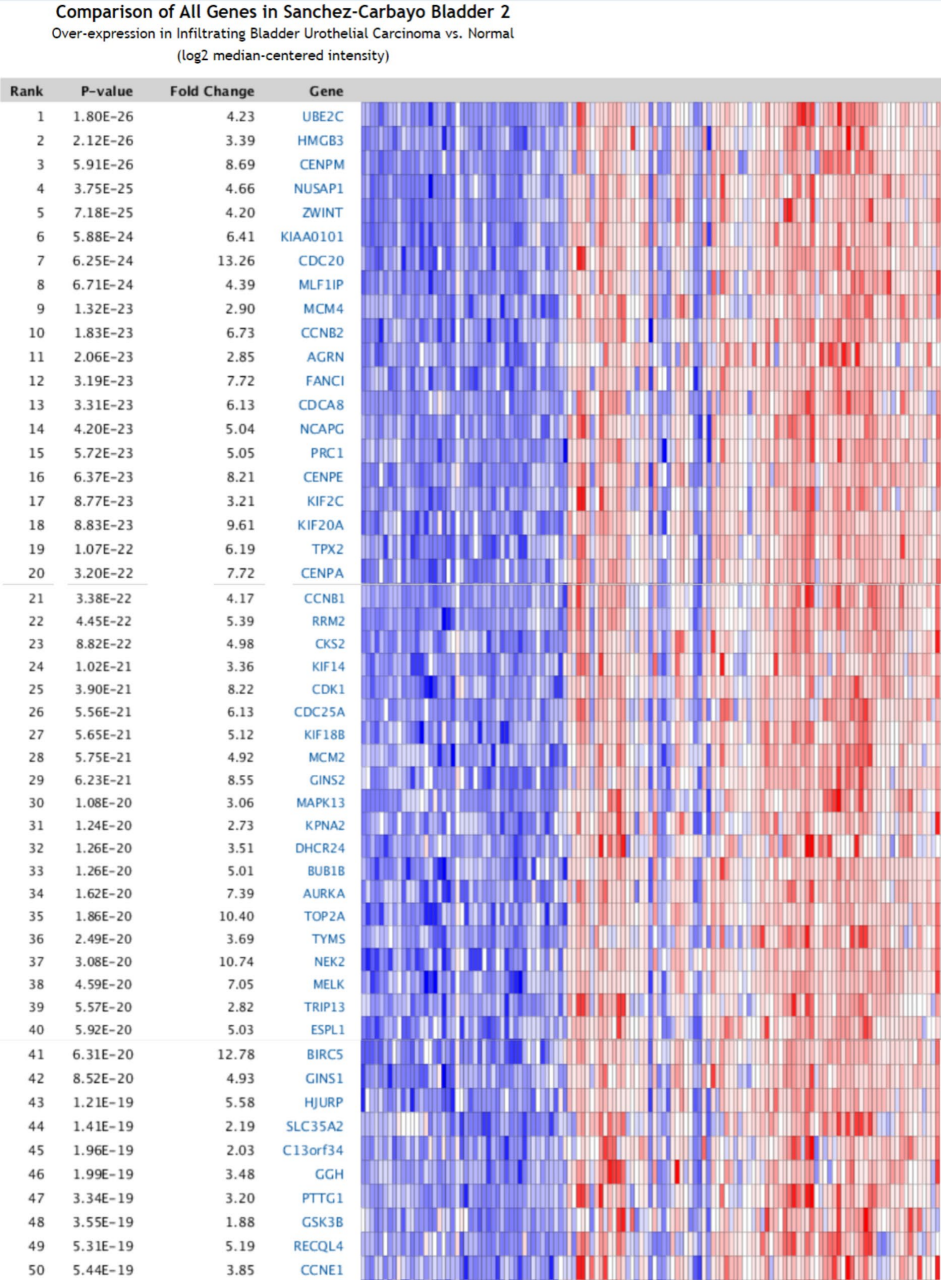
The top 50 over-expressed genes in bladder cancer vs. normal tissue using the Oncomine database

We identified overexpressed genes in BC by comparing BC samples with normal tissues in the Oncomine online database. The top 50 genes overexpressed in 129 BC samples were identified (Fig. 4). The analysis showed that the top 50 genes were mainly enriched in Cell cycle, Oocyte meiosis, Progesterone-mediated oocyte maturation, p53 signaling pathway, Cellular senescence, Human T-cell leukemia virus 1 infection (Table 4).

**Table 4:** KEGG pathways in the top 50 over-expressed genes in bladder cancer vs. normal tissue

term description	count	false discovery rate	Gene ID
Cell cycle	12	1.76E-13	CCNB1, ESPL1, MCM4, CCNE1, MCM2, BUB1B, CCNB2, CDC25A, GSK3B, CDC20, PTTG1, CDK1
Oocyte meiosis	9	6.48E-09	MAPK13, AURKA, CCNB1, ESPL1, CCNE1, CCNB2, CDC20, PTTG1, CDK1
Progesterone-mediated oocyte maturation	6	2.67E-05	MAPK13, AURKA, CCNB1, CCNB2, CDC25A, CDK1
p53 signaling pathway	5	0.00015	CCNB1, CCNE1, CCNB2, RRM2, CDK1
Cellular senescence	6	0.00022	MAPK13, CCNB1, CCNE1, CCNB2, CDC25A, CDK1
Human T-cell leukemia virus 1 infection	6	0.0012	ESPL1, CCNE1, BUB1B, CCNB2, CDC20, PTTG1

### Identification of potential therapeutic target genes of aspirin in bladder cancer

**Fig. 5:**
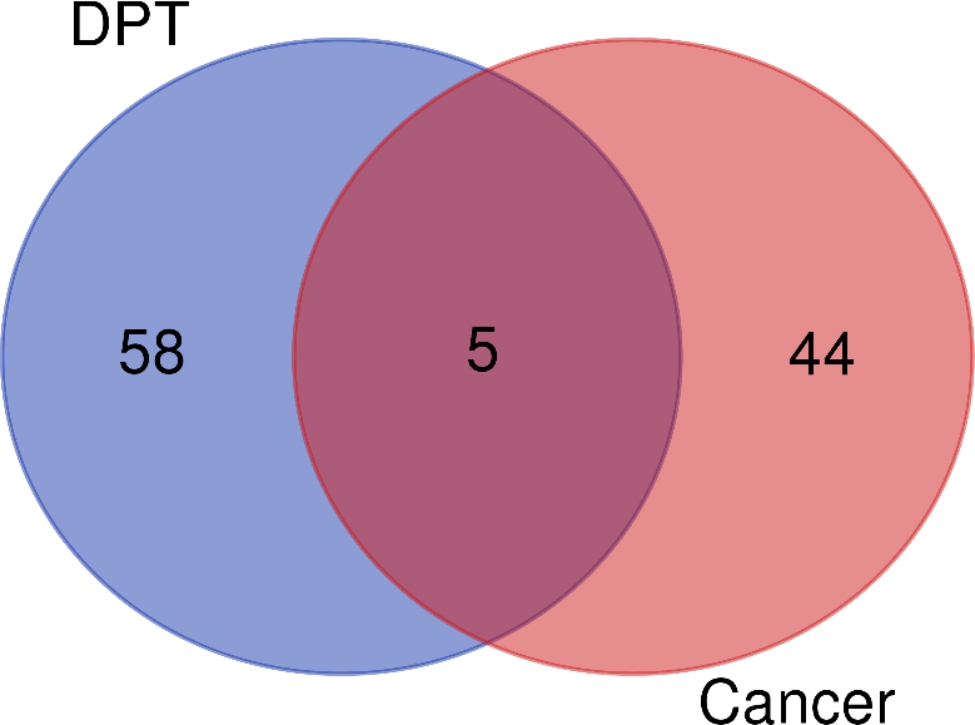
Venn diagram between the overexpressed genes and the three genes associated with aspirin

By comparing the overexpressed genes in BC and the three genes associated with aspirin DPT in BC, we identified five genes GSK3B, CDC20, TPX2, AURKA and CCNE1 (Fig. 5; Table 5) as potential therapeutic targets of aspirin in BC.

**Table 5:** Identification of co-expressed genes from two gene groups

Names	total	elements
DPT(A)	58	CLOCK、COPS5、SUPT3H、CDK4、CDKN1B、CCDC101、RAD52、CCND2、CCGN1、CDKN2C、S100A4、PPARGC1A、USP7、SFN、SKP2、TRRAP、BARD1、TP53、CCND3、CDK6、PTEN、EP300、RCHY1、RPA1、TACC3、SUV39H1、KAT5、CDKN2B、CSTF1、NPM1、RB1、DDX5、TP53BP2、PLK1、MDM2、WRN、SIRT1、SP1、RPL5、FBXW7、MDM4、CNND1、CDK2、UBE2I、MYC、CREBBP、BCL2L1、MAX、ATRIP、NDN、HSP90AA1、TADA3、BIN1、RPA3、CCNA2、KAT2A、ATM、UBE3A
Cancer(B)	44	ESPL1、CDK1、CENPA、NCAPG、MAPK13、TYMS、DHCR24、AGRN、MELK、GGH、MCM2、KPNA2、TRIP13、MCM4、PTTG1、SLC35A2、NEK2、KIF18B、KIF2C、BIRC5、CENPM、KIF20A、RRM2、MLF1IP、ZWINT、CCNB1、GINS2、HMGB3、TOP2A、KIAA0101、NUSAP1、RECQL4、UBE2C、KIF14、CKS2、CENPE、BUB1B、FANCI、C13orf34、CCNB2、HJURP、PRC1、CDC25A、CDCA8
A and B	5	GSK3B、CDC20、TPX2、AURKA、CCNE1

### Analysis of potential target genes for aspirin treatment of bladder cancer

**Fig. 6:**
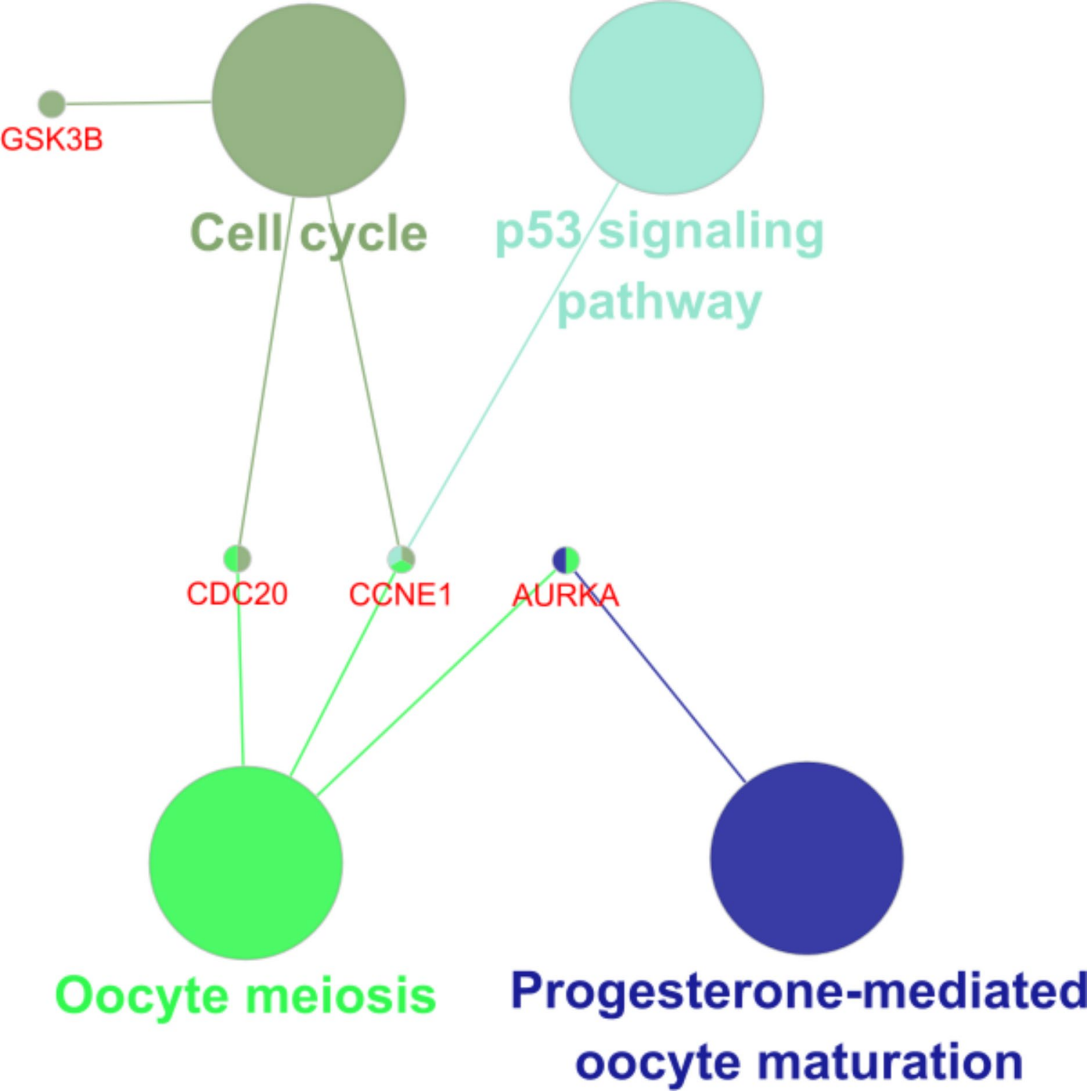
KEGG pathway analysis of aspirin related four predictive targets of bladder cancer

Analysis of the KEGG pathway for these five genes showed that GSK3B, CDC20 and CCNE1 are involved in the cell cycle and AURKA is involved in oocyte meiosis (Fig. 6). Functional annotation of these five genes showed that CDC20 was mainly involved in anaphase-promoting complex-dependent catabolic process, TPX2 was mainly involved in microtubule binding, AURKA was mainly involved in regulation of cytokinesis, CCNE1 was mainly involved in regulation of cyclin-dependent protein serine/threonine kinase activity, and GSK3B was negatively associated with the classical Wnt signaling pathway regulation was associated (Table 6).

**Table 6:** Genetic analysis of potential therapeutic target genes of aspirin in bladder cancer

Gene symbol	Gene name	GO term	Gene function
GSK3B	Glycogen synthase kinase-3 beta	GO:0090090	negative regulation of canonical Wnt signaling pathway
CDC20	Cell division cycle protein 20 homolog	GO:0008054	anaphase-promoting complex-dependent catabolic process
TPX2	Targeting protein for XKLP2	GO:0008017	microtubule binding
AURKA	Aurora kinase A	GO:0032465	regulation of cytokinesis
CCNE1	G1/S-specific cyclin-E1	GO:0000079	regulation of cyclin-dependent protein serine/threonine kinase activity

## Discussion

BC is one of the most common cancers and patients with early or limited BC can be treated by surgical resection, while patients with advanced BC are usually treated with radiotherapy or chemotherapy. Despite effective treatment, the outcome is still unsatisfactory [19]. Bladder cancer is considered to be one of the most frequently mutated cancers in humans, with a mutation rate second only to lung and skin cancers [20, 21]. The major mutation among these mutations is the promoter mutation in the gene encoding telomerase reverse transcriptase (TERT), which occurs at a frequency of 70–80% in bladder cancer patients [22–25]. Therefore, it is essential to find potential therapeutic targets for bladder cancer as soon as possible. And a large number of studies have shown that regular aspirin consumption can reduce the risk of cancer [26–28].

In the present study, we used comprehensive bioinformatics analysis to elucidate the molecular roles of aspirin and its target proteins in BC. First, we analyzed aspirin by [1] identifying the primary DPT of aspirin using Drug Bank [2]. Protein-protein interaction (PPI) networks and signaling pathways of aspirin DPT were analyzed using STRING [3]. Detection and testing of genetic alterations using the cBio portal. [4] Identification of genes associated with 4 aspirin target genes in BC using STRING. We identified 16 action targets of aspirin: PTGS1, PTGS2, AKR1C1, PRKAA1, EDNRA, TP53, HSPA5, RPS6KA3, NFKBIA, TNFAIP6, CASP1, CASP3, CCND1, MYC, PCNA, CCNA2. Among them, CCND1, MYC, and TP53 were associated with BC. Subsequently, we determined that the alteration of CCND1 showed gene amplification. the alteration of MYC mainly showed gene amplification. the alteration of TP53 mainly included missense mutation, splice mutation and truncation mutation. finally, we constructed a PPI pathway consisting of three target genes of BC to predict the potential target genes of aspirin in BC. Meanwhile, we identified the top 50 overexpressed genes of BC using Oncomine. Finally, we identified the co-expressed genes (GSK3B, CDC20, TPX2, AURKA and CCNE1) among the genes interlinked with the 3 aspirin target genes in BC samples as potential targets for aspirin treatment of BC.

GSK-3β, GSK3B, is a positive regulator of NF-κB transcriptional activity [29, 30]. It has been shown that NF-κB plays a role in human cancer progression and chemoresistance [31, 32] through positive regulation of its target genes XIAP [33] and Bcl-2 [34].Levidou et al. [32]showed that nuclear expression of NF-κB correlates with histological grading and staging of bladder cancer.Sei Naito et al. found that urothelial epithelial carcinoma cells and abnormal nuclear accumulation of GSK-3β in most human bladder cancers. nuclear expression of GSK-3β was associated with high malignancy, metastasis and poorer survival in bladder cancer patients. They suggested that GSK-3 is a positive regulator of bladder cancer cell proliferation and survival [35]. cdc20 is usually considered as an oncogenic factor that promotes tumor development [36, 37]. Moreover, it has been demonstrated that increased CDC20 expression in bladder cancer patients is associated with poor prognosis [38].AURKA and AURKB, of the AURKA kinase family, are closely associated with the development of malignancy.AURKA is a cell cycle-associated serine-threonine kinase that is overexpressed in various types of cancer and is strongly associated with poor prognosis [39].Mobley et al. [40] found that knockdown AURKA had little effect on bladder cancer cell proliferation but prevented tumor cell invasion, and that overexpression of AURKA was associated with poor prognosis.AURKB is a key regulator of malignant mitosis and is involved in chromosome segregation and cytoplasmic division.Bufo et al. [41] found that high expression of AURKB may be involved in bladder carcinogenesis and hypothesized that bladder cancer could be treated in the future by targeting AURKB expression and specific antimitotic agents. dysregulation of CCNE1/2 activity is present in various cancers [42–45], leading to disruption of the G1-S transition and uncontrolled cell proliferation. involvement of the CCNE-CDK2 complex in cell cycle regulation has been demonstrated to play an important role in tumor development [46, 47]. the E2F transcription factor strongly activates CCNE1 and CCNE2, the CCNE-CDK2 complex phosphorylates and inactivates Rb, and phosphorylated Rb releases the E2F transcription factor, thus promoting cell cycle progression from G1 to S phase [48]. In addition, it has been demonstrated that MNX1 induces bladder cancer proliferation and tumorigenicity by targeting promoters to upregulate CCNE1 and CCNE2 expression [49].

As for the TPX2 gene, by String prediction, we learned that no complete biological process, signaling pathway related to TPX2 has been found so far. However, there are many literatures that have demonstrated that TPX2 is significantly associated with bladder cancer. liang Yan et al. demonstrated that overexpression of TPX2 promotes bladder cancer growth, while overexpression of GLIPR1 or p53 inhibits bladder cancer growth. Increasing evidence supports the role of TPX2 as a tumor promoter in human tumor development, with bladder cancer tissues expressing high TPX2 levels having upregulated p53 expression and downregulated GLIPR1 expression. In addition, TPX2 and p53 expression was lower in non-muscle-infiltrating bladder cancer cells than in muscle-infiltrating bladder cancer cells, while the opposite pattern of GLIPR1 expression was observed [50]. overexpression of GLIPR1 suppressed TPX2. meanwhile, SP1 and c-Myb expression were negatively correlated with GLIPR1 expression. [51–53]. Yan et al. demonstrated that TPX2 is highly expressed in human bladder cancer tissues and that upregulation of TPX2 predicts poor prognosis in patients with bladder cancer. In addition, TPX2 promotes T24 cell proliferation and tumorigenesis and blocks apoptosis [54].

In conclusion, we believe that these five target genes (GSK3B, CDC20, TPX2, AURKA and CCNE1) may promote the occurrence of bladder cancer and lead to poor prognosis.We explored the potential therapeutic targets of aspirin for bladder cancer by comprehensive bioinformatics analysis. We suggest that aspirin acts through cell cycle and signaling pathways in bladder cancer cells, and our results aim to provide new clues to elucidate the mechanisms of aspirin’s action in bladder cancer.

However, there are still challenges in applying Web-based data to the study of drugs such as aspirin. Identifying drug-target interactions is important in the drug discovery process. Although microarrays, proteomics and other high-throughput screening analyses have been applied, experimental methods for drug-target interaction identification remain challenging.

## Conclusion

In short, comprehensive bioinformatics analysis provides researchers with a simple and convenient method that can use existing drug information and cancer genetic changes as a guide for testing hypotheses and help researchers apply basic research to the clinic.

## Data Availability

All data generated or analyzed during this study are included in this published article.

## List of abbreviations

(BC): bladder cancer.
NMIBC: Non-muscle invasive bladder cancer.
PD-L1: Programmed death receptor ligand 1.
KEGG: Kyoto Encyclopedia of Genes and Genomes.
DPTs: Protein targets.
PPI: Protein-protein interaction.
